# Changes of Ocular Surface and the Inflammatory Response in a Rabbit Model of Short-Term Exposure Keratopathy

**DOI:** 10.1371/journal.pone.0137186

**Published:** 2015-09-03

**Authors:** Chun-Ting Lai, Wei-Chieng Yao, Szu-Yuan Lin, Hsin-Yu Liu, Huai-Wen Chang, Fung-Rong Hu, Wei-Li Chen

**Affiliations:** 1 Department of Ophthalmology, National Taiwan University Hospital, Taipei, Taiwan; 2 Department of Anesthesia, Min-Sheng General Hospital, Tao-Yuan City, Taiwan; 3 Deparment of Ophthalmology, Cathay General Hospital, Taipei, Taiwan; 4 Center of Corneal Tissue Engineering and Stem Cell Biology, National Taiwan University Hospital, Taipei, Taiwan; Wayne State University, UNITED STATES

## Abstract

**Purpose:**

To evaluate the ocular surface change and the inflammatory response in a rabbit model of short-term exposure keratopathy.

**Methods:**

Short term exposure keratopathy by continuous eyelid opening was induced in New Zealand white rabbits for up to 4 hours. Ultrasound pachymetry was used to detect central total corneal thickness. In vivo confocal microscopy and impression cytology were performed to evaluate the morphology of ocular surface epithelium and the infiltration of inflammatory cells. Immunohistochemistry for macrophage,neutrophil, CD4(+) T cells, and CD8(+) T cells were performed to classify the inflammatory cells. Scanning electron microscopy(SEM) was performed to detect ocular surface change.The concentrations of IL-8, IL-17, Line and TNF-αwere analyzed by multiplex immunobead assay. TUNEL staining was performed to detect cellular apoptosis.

**Results:**

Significant decrease ofcentral total cornealthickness were found within the first 5 minutes and remained stable thereafter, while there were no changes of corneal epithelial thickness.No significant change of corneal, limbal and conjunctival epithelial morphology was found by in vivo confocal microscopy except the time dependent increase of superficial cellular defects in the central cornea. Impression cytology also demonstrated time dependent increase of sloughing superficial cells of the central cornea. Aggregations ofinflammatory cells were found at 1 hour in the limbal epithelium, 2 hours in the perilimbal conjunctival epithelium, and 3 hours in the peripheral corneal epithelium.In eyes receiving exposure for 4 hours, the infiltration of the inflammatory cells can still be detected at 8 hours after closing eyes.Immunohistochemical study demonstrated the cells to be macrophages, neutrophils, CD4-T cells and CD-8 T cells.SEM demonstrated time-depending increase of intercellular border and sloughing of superficial epithelial cells in corneal surface. Time dependent increase of IL-8, IL-17 and TNF-α in tear was found.TUNEL staining revealed some apoptotic cells in the corneal epithelium and superficial stroma at 3 hours after exposure.

**Conclusions:**

Short term exposure keratopathy can cause significant changes to the ocular surface and inflammatory response. Decrease of central total corneal thickness, aggregation of inflammatory cells, and cornea epithelial cell and superficial keratocyte apoptosis were found no less than 4 hours following the insult.

## Introduction

The superficial part of the cornea is a layer of stratified, non-keratinized, non-secretory epithelium, whichrelies on a stable tear film to maintain healthy corneal physiology and optical properties.[1–3]Any pathological conditions causing dry eye disease may result in discomfort, visual impairment, and tear film instability with the potential to damage ocular surface.[4–6]Among the etiologies of dry eye syndrome, exposure keratopathy is uniquely caused by not only an unclosed eyelid, but also incomplete blinking.[7–10]Eyelidclosure and blinking contribute to replenishing and spreading the tear film across the corneal surface and preventing tear film evaporation. Exposure keratopathy can be classified into neurotrophic (cranial nerve V palsy, aneurysm, cerebrovascular accident, multiple sclerosis, tumor, herpes simplex, herpes zoster),[11–13] neuroparalytic (cranial nerve VII palsy),[14] lid malposition(lagophthalmos, proptosis),[15] and iatrogenic event(general or topical anesthesia, ocular procedure or surgery, artificial respiration in anintensive care unit).[16, 17]Exposure keratopathy may lead to chemosis, cornea erosion, corneal melting, infectious keratitis, and even corneal perforation.[18–20] In terminal patients willing to donor corneas, the protection of corneal damage from exposure keratopathy is also important to assure acceptable quality of donor corneas.[21–23]During excimer laser refractive surgery, iatrogenic exposure keratopathy may significantly affect the surgical results.[24, 25]

Although numerous dry eye studies having been conducted, most of the studies focused on dry eye secondary to aqueous tear deficiency,[26–31]or animal model of desiccating stress[32–36]. The role of inflammation on the pathogenesis of aqueous tear deficientdry eyehas drawnresearch attention during the past few years, and thus anti-inflammatory agents have been proposed to treat dry eye syndrome in conjunction with the use of artificial tears.[37, 38]As for evaporative type of dry eye, most of the studies focusing on desiccating stress (with or without inhibition of tear secretion) in mouse eyes which were exposed to a controlled environment chamber with stable humidity and temperature. In those studies, continuous desiccating stress was applied for about 10 hours a day for more than 1 week and the experimental mice can blink their eyes freely. In addition, the exposure to a continuous air draft from a fan placedin front of the cageis usually performed[32–36, 39]. None of the procedures was similar to the pathogenic condition in which patients with exposure keratopathy encountered.It's worthy to building up ananimal model to mimic exposure keratopathy in which theblinking and closure of eyes are impaired,and the ocular surface dried out in natural condition for a period of time. Such experimental condition can be applied to patients with exposure keratopathy, especially for patients in intensive care units or operative rooms if the ocular surface is not adequately protected.

In the current study, we built up a rabbit model of short term exposure keratopathy. *In vivo* confocal microscopy, immunohistochemistry, ultrasound pachymetry, impression cytology,scanning electron microscopy (SEM) and TUNEL staining were performed to identify the early changes of the cornea, limbus, and conjunctiva after the induction of exposure keratopathy. Our results demonstrated the significant changes of ocular surface in early exposure keratoapthy,which have not been previously reported.

## Materials and Methods

### Animals models of short-term exposure keratopathy

New Zealand albino rabbits (female, weight, 3.0–3.5 kg; age, 6 months) were used in this study. Use, care, and treatment of all animals followed the regulations of the ARVO Statement for the Use of Animals in Ophthalmic and Vision Research. All experimental procedures were approved by the Committee for Animal Research of the National Taiwan University Hospital. All in vivo experimental procedures (including pachymetry, in vivo confocal microscopy and impression cytology) were performed under general anesthesia induced by the intramuscular injection of ketamine hydrochloride (35 mg/kg) and xylazine hydrochloride (5 mg/kg). The model of exposure keratopathy was modified by other studies of desiccating stress[32–36]. In brief, the experiments were performed in an environmentally controlled room (50–60%humidity, 25 to 28°C). The right eyes of the animals were used for all experiments, and the left eyes were left untreated. Eyelid specula were appliedon the right eyes continuously without adding any lubricants. The interpalpabral fissures were maintained widely open to assure the exposure of central cornea, limbus and perilimbal conjunctivafor following time periods: every 5 minutes in the first 30 minutes and hours 1, 2, 3, and 4. After exposure for 4 hours, eyelid specula were removed and rabbit eyes were maintained closed for 4, 8 and 24 hours for recovery. For immunohistochemistry and SEM, rabbits at different time points (post-exposure for 3 hours for immunohistochemistry and post-exposure 1, 2 and 4 hours for SEM) were euthanized with intravenous injection of 240 mg/kg thiamylal sodium (Shinlin Singseng Pharmaceutical, Taoyuan, Taiwan). Six eyes were included for each time point.

### Central total corneal thickness measured by ultrasound pachymetry

To measure the central total corneal thickness, ultrasound pachymetry (OcuScan, Alcon, Fort Worth, TX) was performed on the center of the cornea. To prevent the artifacts caused by topical medications, the cornea remained dry during the examination without adding topical anesthetic medications or lubricants.

### Observation of corneal epithelial condition under surgical microscopy

At different time points, topical fluorescein dye was applied on corneal surface, and the severity of any corneal epithelial defect was recorded under surgical microscopy illuminated with cobalt blue light (OPMI Pico I; Carl Zeiss Meditec, Jena, Germany).

### *In vivo* confocal microscopy

*In vivo* confocal microscopy was performed on the right eye of all subjects at different time points with the Heidelberg Retinal Tomography(HRT-3) equipped with a Rostock Corneal Module (RCM) (Heidelberg Engineering GmbH, Heidelberg, Germany). This instrument uses a 60× water-immersion objective lens (Olympus Europa GmbH, Hamburg, Germany) and a 670 nm diode laser as a light source, resulting in an image dimension of 400 × 400 μm^2^ and a transverse resolution of 1 μm.

**Fig 1:**
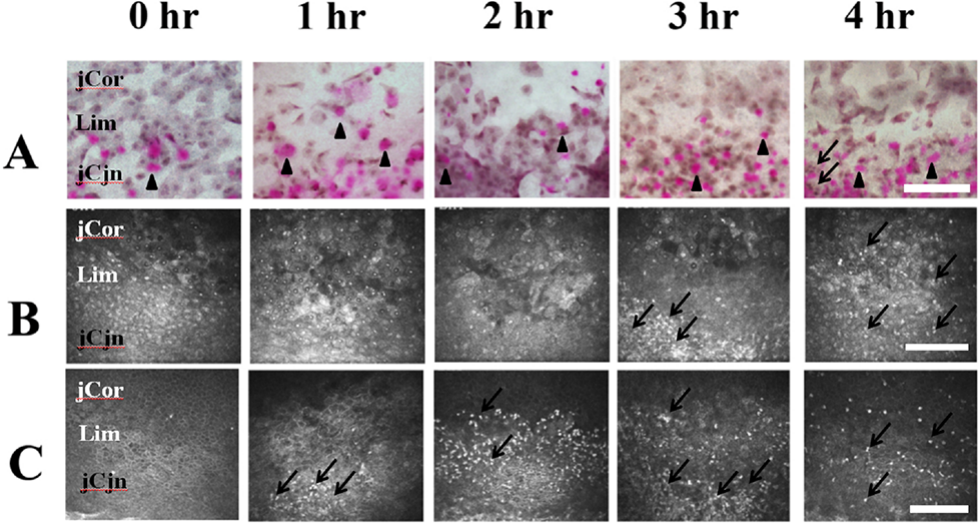
Impression cytology and in vivo confocal microscopy of the limbal epithelium. (A) Impression cytological findings. At post exposure 1 to 4 hours, the morphology of the superficial epithelial cells and goblet cells (black arrow heads, the purple dots indicate goblet cells containing mucin which were stained by PAS) attached to the filter papers were similar. At post exposure 4 hours, some inflammatory cells (black arrows) can be found at the juxta-limbal conjunctival site. (B) In vivo confocal microscopic findings of the surface epithelial layer of the limbus. there was no significant change of morphology of superficial cells from post exposure 1 to 4 hours. An aggregation of inflammatory cells (black arrows) can be found atpost exposure 3 hours to 4 hours. (C) In vivo confocal microscopic findings of the basal epithelial layer of the limbus. There was no significant change of basal cell morphology from post exposure 1 to 4 hours. Aggregation of inflammatory cells (black arrows) can be found at post exposure 1 and 2 hours. The inflammatory cells migrated to the juxta-limbal corneal and juxta-limbal conjuncival areas at post-exposure 3 and 4 hours.jCor: juxtal-limbal cornea. Lim: limbus. jCjn: juxta-limbal conjunctiva. hr: Hours after exposure. Bar: 200 um.

Before examination, one drop of vidisic gel (HanBul Pharm Co., Ltd., Seoul, Korea) was applied to the surface of a sterile disposable plastic cap (Tomo-cap, Heidelberg Engineering GmbH, Heidelberg, Germany) on the front lens of the microscope. The surface of the tomo-cap was positioned on the following 3 locations: (1)central cornea, (2)limbus, and (3)bulbar conjunctiva 2 mm from the limbus.In the limbal images, the corneal part shown as the upper 1/3 of the image (Fig 1A) was termed as juxta-limbal cornea, and the conjunctical part shown as the lower 1/3 of the image (Fig 1A) was termed as juxta-limbal conjunctiva in this study.The images were examined layer by layer. The scanning was performed continuously from the anterior surface of the cornea to the posterior surface. At least 6 examinations per eye were performed.

### Impression cytology

Impression cytology was performed using a mixed cellulose ester membrane filter with a pore size of 0.22 μm (Millipore, MA). The membrane was applied to the following locations of ocular surface, including the central cornea, limbus, and bulbar conjunctiva 2 mm from the limbus. After being removed andfixed with 95% ethanol, all specimens were stained with periodic acid-Schiff (PAS),counterstained with hematoxylin (Sigma Aldrich, St. Louis, MO), and mounted with Permount. Cytology features were observed by Eclipse E800 Nikon Microscope equipped with a Spot Digital Camera and Spot version 1.1 CE software (Diagnostic Instruments, Sterling Heights, MI) at a magnification of ×200.

### Immunohistochemistry

Rabbit eyes were cryopreserved, cut into 8-μm sections, air-dried and fixed in 4% paraformaldehyde for 10 min. Sections were then permeabilized with 0.4% Triton X-100, and blocked with bovine serum albumin with bovine serum. The infiltration of macrophageswere evaluate with mouse monoclonal anti-human AM-3K antibody (Cosmo Bio Co. Ltd, Tokyo, Japan).[40–42]The infiltration of neutrophils was demonstrated with mouse anti-rabbit neutrophil antibody (NIMP-R14, ab2557, Abcam,Cambridge, UK). Monocloncal rat anti-mouse neutrophil antibodies (Thermo Fisher Scientific Inc., Waltham, MA, USA) were used to detect the infiltration of CD4- and CD-8 positive T lymphocytesseparately.Terminal deoxynucleotidyl transferase dUTP nick end labeling (TUNEL) staining kit was purchased from Biovision (Milpitas, CA), and thestaining was used to evaluate the apoptosis of ocular surface cells.The staining pattern of the tissue sections was observed by conventional fluorescence microscopy using an Eclipse E800 Nikon Microscope with a VFM Epi-Fluorescence Attachment (Nikon, Melville, NY) equipped with a Spot Digital Camera and Spot version 1.1 CE software (Diagnostic Instruments, Sterling Heights, MI). All experiments were repeated 6 times to ensure consistent results.

### Scanning Electron Microscopy (SEM) Observation

At different time points after exposure, the rabbit corneas were obtained after sacrifice. The corneas were carefully excised, fixed in 4% glutaraldehyde in 0.05 M cacodylate buffer for 1 hour, and then postfixed in 1% osmium tetroxide in veronal acetate buffer containing 0.22 M sucrose. The fixed materials were dehydrated through a series of ethanol washes. The corneas were placed in t-butyl alcohol, treated in a freeze-drying apparatus (EIKO ID-2; EIKO, Tokyo, Japan), and then sputter coated with gold using an auto fine coater (JEOL JFC-1600; JEOL, Tokyo, Japan). After processing, the surface of the corneal epithelium was observed by means of a SEM microscope (Hitachi SU8220; Hitachi, Ibaragi, Japan).

### Measurement of IL-8, IL-17 and TNF-apha level in tear

To collect tear sample from pre-exposure (exposure 0 hour, control) and post-exposure 4 hours, 30 ul of phosphate-buffered saline was instilled into the inferior fornix. We collected 20 ul of tear fluid and buffered by micropitte at the medical and laterior canthus. To minimize ocular surface irritation, we collected the mixture as soon as possible after phosphate-buffered saline instillation. The fluid was place into a 1.5 mL Eppendorf tube and stored at-70°C until further examination. The concentration of the cytokines was measured using Quantibody Rabbit Cytokine Array (Catalog#: QAL-CYT-1, RayBiotech, Norcross, GA)

### Statistical Analysis

For central total corneal thickness and corneal epithelial thickness, experimental data were analyzed using Student’s two tailed t-test. To compare the cytokine level in tears, the data were analyzed using Wilcoxon signed-rank test. All the results were expressed as the mean± standard deviation. The probability of p<0.05 was considered to be statistically significant.

## Results

### 1. Corneal epithelial defects

**Fig 2:**
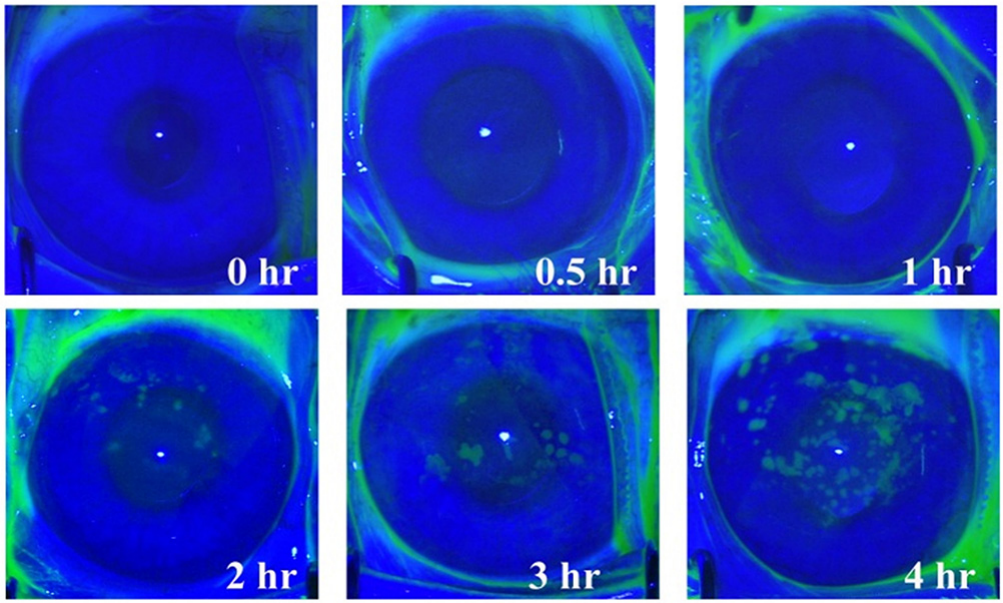
The external eye photography with fluorescein staining revealed grossly detectable epithelial defect(>0.3×0.3 mm^2^)firstly at 2 hours after exposure. The number of the defect areas increased time-dependently.

Corneal epithelial defects (>0.3×0.3 mm^2^ per defect area) were detected starting 2 hour post-exposure, and time-dependently increased to 4 hours after exposure (Fig 2).

### 2. Change of the central total corneal thickness

**Fig 3:**
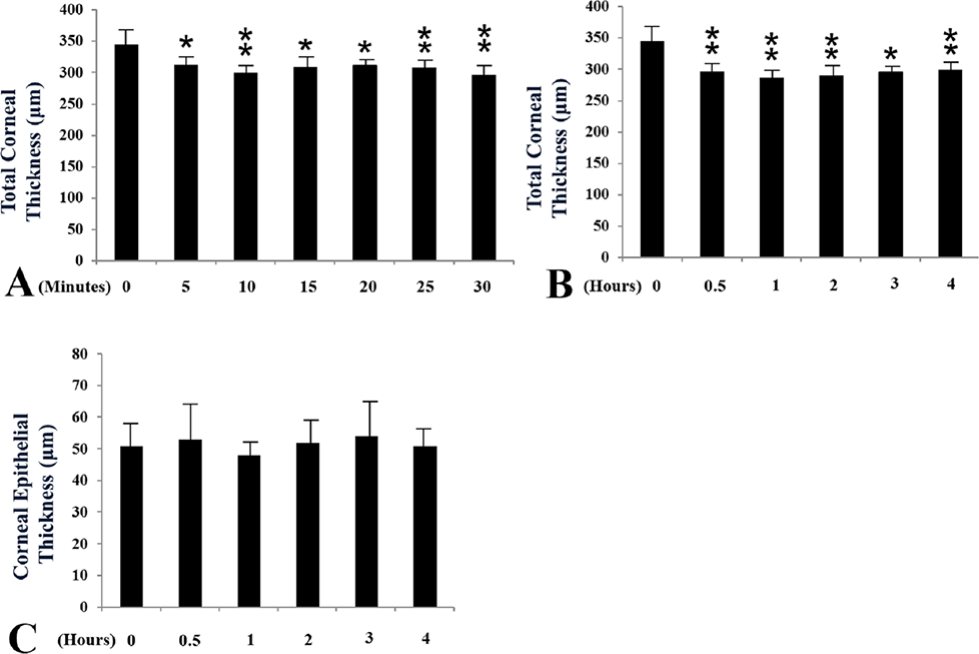
The changes of central total corneal and corneal epithelial thickness after exposure. (A,B) The changes of central totalcorneal thickness from post exposure 0 to 30 minutes (A) and 0 to 4 hours (B). (C) The changes of central corneal epithelial thickness from post exposure 0 to 4 hours. * and ** indicated significant differences compared to the pre-exposure group (*: p<0.05; *: p<0.01 by Student’s*t* test).

The central total corneal thickness, evaluated by ultrasound pachymetry, decreased dramatically from 0 minutes (342.3 ±11.2μm) to 5 minutes (318.7±12.5μm) (*P*<0.05)post-exposure (Fig 3A). However, the central corneal thickness remained stable from 5 minutes until the end of 4 hours post-exposure (Fig 3A and 3B). Corneal epithelial thickness, evaluated by *in vivo* confocal microscopy, demonstrated the stable thickness during the entireobservational period (*P*>0.05) (Fig 3C).

### 3.Change of the central cornea

#### Impression cytology

**Fig 4:**
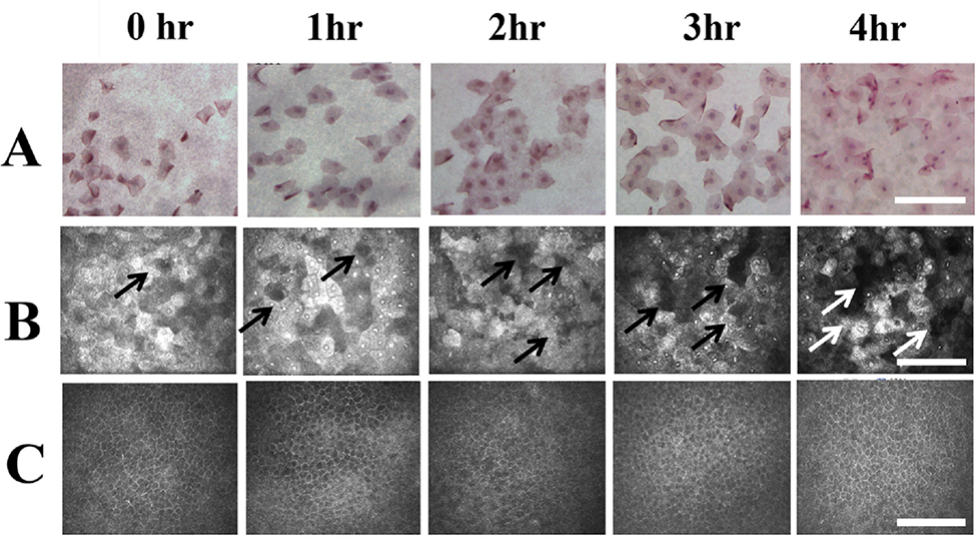
Impression cytology and in vivo confocal microscopy of the central cornea epithelium. (A) Impression cytological findings. The longer time the cornea exposure, the more surface cells attached to the filtering paper were found. However, the morphology of the detached cells remained similar from post-exposure 1 hour to 4 hours. (B). In vivo confocal microscopic findings of the surface epithelial layer of the central cornea. From 1 hour to 4 hours after exposure, the time dependent increase of cellular disappearance (White and black arrows) was found, which indicated the sloughing of the cell. (C) In vivo confocal microscopic findings of the basal epithelial layer of the central cornea. There was changes of cellular morphology or density from post-exposure 1 to 4 hours. During the observational period, there was no inflammatory cells detected at the superficial or the basal epithelial layers. hr: Hours after exposure. Bar: 200 um.

From 1 hour to the end of 4 hours after exposure to the dessicating stimulus, the number of the superficial corneal epithelial cells attached to the filtering papers progressively increased. However, the cells retained their normal squamous morphology. Furthermore, no inflammatory cells were detected (Fig 4A).

#### *In vivo* confocal microscopy

From 1 hour to 4 hours after exposure, the localized loss of superficial epithelial cells, which indicated the sloughing of the cell, was detected (Fig 4B). However, there was no obvious morphological changes in the superficial epithelial cells during the whole observational period. The morphology and density of corneal basal cells remained stable from the beginning to 4 hours after exposure (Fig 4C). There was no inflammatory cells detected until the end of the experiment.

### 4. Change of the limbus

#### Impression cytology

From post-exposure 1 hour to the end of 4 hours, the morphology of the superficial epithelial cells and goblet cells attached to the filter papers remained constant (Fig 1A). Sporadically distributed inflammatory cells were found in the juxta-limbal conjunctiva at 4 hours post exposure (Fig 1A), which is consistent with the finding from in vivo confocal microscopy(Fig 1B).

#### *In vivo* confocal microscopy

In the limbus, there was no significant change of morphology of superficial and basal cells from post exposure 1 to 4 hours (Fig 1B and 1C). An aggregation of inflammatory cells was initially detected 1 hour post exposure at the basal limbal epithelial layer, then increased in number and spread to the juxta-limbal basal conjunctiva at post-exposure 2 hours. At post exposure 3 hours, the inflammatory cells at the basal limbal epithelial layer migrated more extensively to the juxta-limbal basal corneal epithelium and juxta-limbal basal conjunctival epithelium, and can be detected at the juxta-limbal conjunctival superficial epitheliums. At post exposure 4 hours, the aggregation phenomenon of the inflammatory cells disappeared, and the cells spread to the junxta-limbal cornea and juxta-limbal conjunctiva in both the superficial and basal epithelial layers (Fig 1B and 1C).

### 5. Change of the peripheral conjunctiva at 2 mm away from the limbus

#### Impression cytology

**Fig 5:**
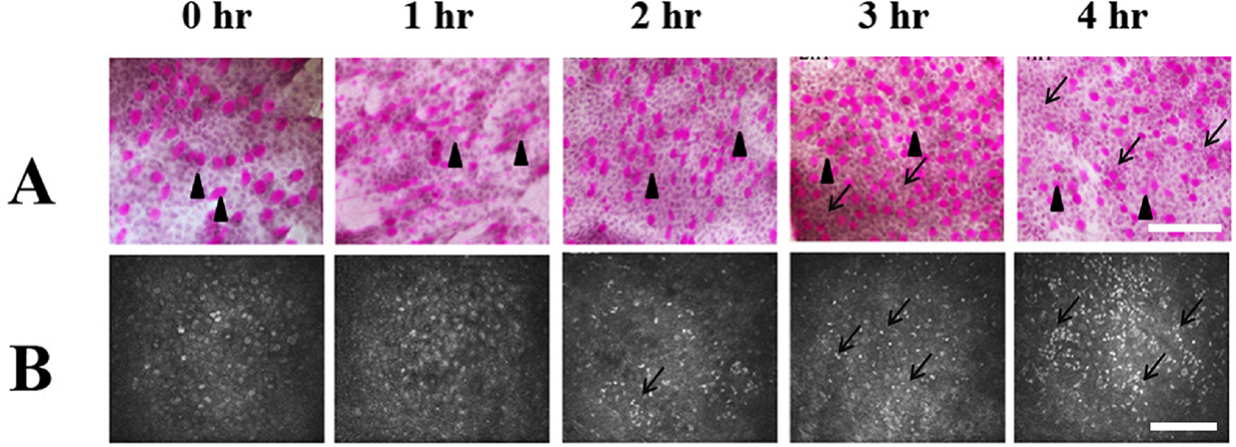
Impression cytology and in vivo confocal microscopy of the peripheral conjunctval epithelium at 2 mm from the limbus. (A) Impression cytological findings. At post exposure 1 to 4 hours, the morphology of the superficial conjunctival epithelial cells and goblet cells (black arrow heads, the purple dots indicate goblet cells containing mucin which were stained by PAS)attached to the filter papers were similar. Abundant inflammatory cells were found from at post exposure 3 and 4 hours ((black arrows). (B) In vivo confocal microscopic findings of the surface epithelial layer of the conjunctiva. There was no significant change of morphology of superficial cells from post exposure 1 to 4 hours. No goblet cells shown by impression cytology was clearly identified. A time dependent increase of inflammatory cells (black arrows) can be found at post exposure 2, 3 and 4 hours. hr: Hours after exposure.Bar: 200 um.

From post-exposure 1 hour to the end of 4 hours, the morphology and density of the conjunctival epithelial cells and goblet cells remained stable. However, numerous inflammatory cells can be detected from at post exposure 3 and 4 hours. (Fig 5A).

#### *In vivo* confocal microscopy

From 1 hour to the end of 4 hours post exposure, the number and morphology of the superficial conjunctival cells remained constant. However, time dependent increase of inflammatory cells were found from post exposure 2 to 4hours (Fig 5B).

### 6. Change of the inflammatory reaction during the recovery process

**Fig 6:**
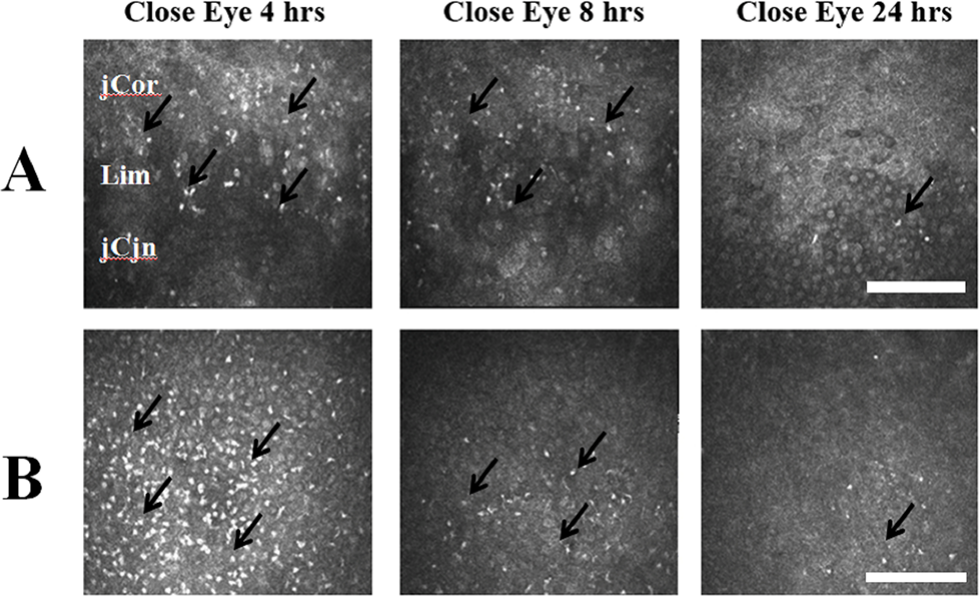
Time dependent change of inflammatory cellular infiltration after termination of eye exposure by in vivo confocal microscopy. The eye closure (termination of eye exposure) was performed on eyes undergoing exposure for 4 hours.(A) Detecting on the limbus. After termination of eye exposure for 4 hours and 8 hours, still abundant inflammatory cells can be detect. After termination for 24 hours, only minimal inflammatory cells can be traced. (B) Detecting on the peripheral conjunctval epithelium at 2 mm from the limbus. After termination of eye exposure for 4 hours, abundant inflammatory cells can be detect. Time dependent decrease of inflammatory cells was found at 8 hours and 24 hours after termination of the eye exposure.Cor: juxtal-limbal cornea. Lim: limbus. jCjn: juxta-limbal conjunctiva. hr: Hours after exposure. Black arrows: infiltration of inflammatory cells. Bar: 200 um.

In vivo confocal microscopy showed time dependent change of the inflammatory cellular infiltrate at the recovery phase after exposure for 4 hours (Fig 6). The strong infiltration of the inflammatory cells was still detected at 4 hours and 8 hours after termination of the eye exposure (Fig 6A and 6B). At 24 hours after the termination of eye exposure, only minimal infiltration of the inflammatory cells was detected (Fig 6C).

### 7. Immunohistochemistry and TUNEL staining

**Fig 7:**
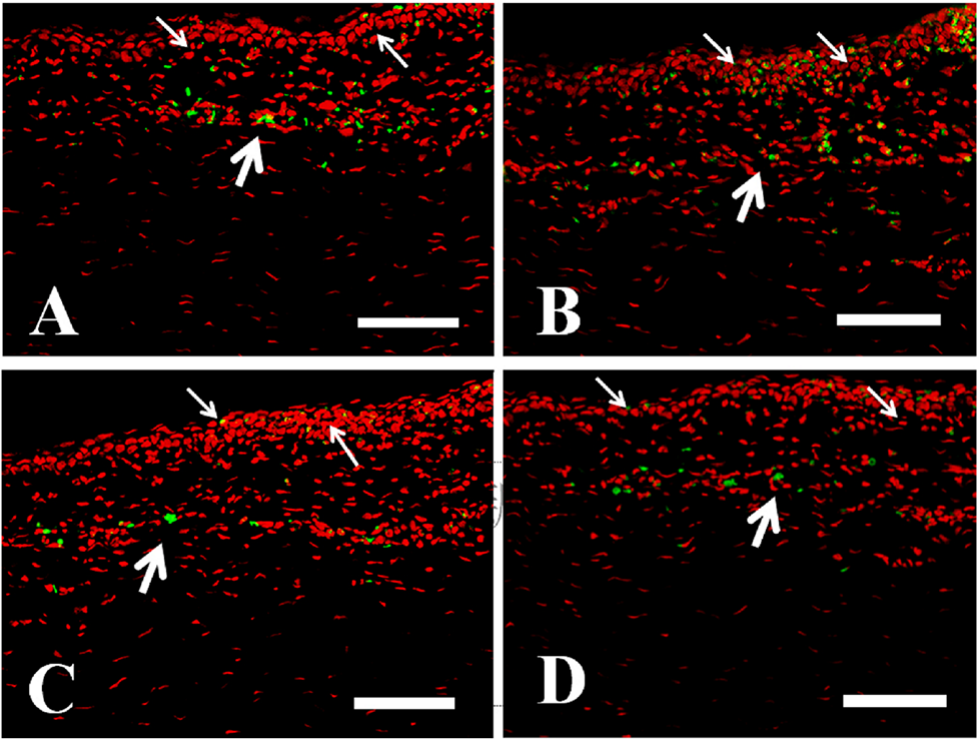
Immunohistochemical staining at the limbus at post-exposure 3 hours. (A) AM3K cells, which indicated macrophages, can be found in the epithelial layer (white thin arrows) and the superficial stroma (white thick stroma). (B) Neutrophils can be found in the epithelial layer (white thin arrows) and the superficial stroma (white thick stroma). (C)CD4 (+) cells, which indicated T helper cells, can be found in the epithelial layer (white thin arrows) and the superficial stroma (white thick stroma). (D) CD 8(+) cells, which indicated T killer cells, can be found in the epithelial layer (white thin arrows) and the superficial stroma (white thick stroma). Green: the staining of macrophages, neutrophils, CD4+ and CD8+ T cells. Red: PI for staining of nucleus. Scale bar: 50 um.

**Fig 8:**
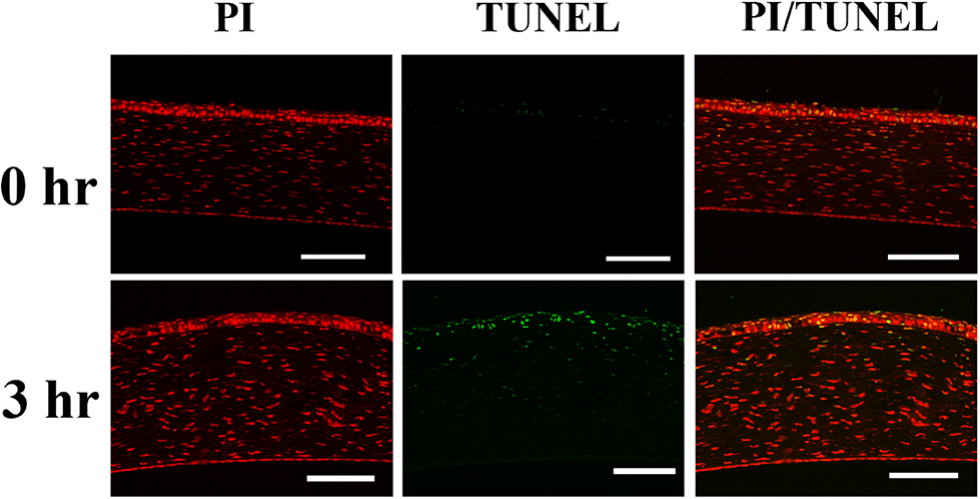
TUNEL staining for the detection of apoptotic cells in the central cornea. At post exposure 3 hours, the TUNEL stain positive cells can be detected in the corneal epithelium and superficial keratocytes. Green: TUNEL, which stains apoptotic cells. Red: PI, which stains nucleus. Scale bar: 100 um. hr: Hours after exposure.

Macrophages, neutrophils, CD4(+) T-cellsand CD8(+) T-cells were found on the epithelial layers and subepithelial stromal layers of the limbus, peri-limbal cornea, and perilimbal conjunctivaat post-exposure 3 hours (Fig 7). The result is consistent withinflammatory cell infiltration detected by *in vivo* confocal microscopy.The epithelial layer of central cornea, showed positive TUNEL staining at post exposure 3 hours (Fig 8) and 4 hours (data not shown).

### 8. Scanning Electron Microscopy (SEM) observation

**Fig 9:**
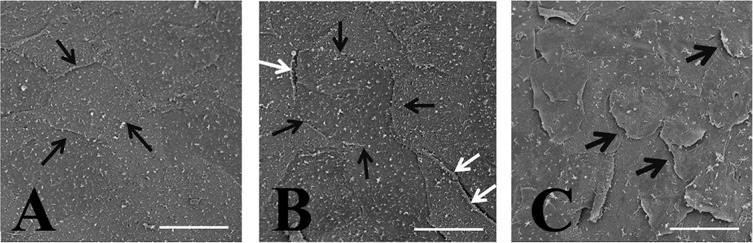
SEM examination on corneal epithelial cells at different time points after exposure (A) At post-exposure 1 hour, no obvious superficial epithelial sloughing, or increased intercellular gaps was found. (B) At post-exposure 2 hours, the increased intercellular gaps can be clearly seen. However, no obvious epithelial sloughing can be detected. (C) At post-exposure 4 hours, sloughing of superficial epithelial cells were observed. Black thin arrow: intercellular borders. White arrows: the increased intercellular borders with mild detachment of the epithelial cells from underlying layers. Black thick arrows: Sloughing of the superficial epithelial cells from the underlying layers. Bar: 50 um”

At different time points after exposure, the SEM showed time-dependent increase of intercellular gaps in corneal superficial epithelial layer (Fig 9). At post-exposure 1 hour, no obvious superficial epithelial sloughing, or increased intercellular gaps was found (Fig 9A). At post-exposure 2 hours, the increased intercellular gaps can be clearly seen (Fig 9B). At post-exposure 4 hours, sloughing of superficial epithelial cells were observed (Fig 9C)

### 9. Expression of IL-8, IL-17 and TNF-αlevel in tear

Table 1 demonstrated the expression level of tear cytokines, including IL-8, IL-17 and TNF-αin different exposure conditions. The tear IL-8, level was 71.3±10.3 pg/ml,381.5±109.1pg/ml and 631.1±147.5 pg/ml in the control (exposure 0 hour), exposure 2 hours and exposure 4 hours groups, respectively. The tear IL-17 level was 356.2±43.4 pg/ml, 342.6±51.6 pg/ml, and 643.5±159.7pg/ml in the control, exposure 2 hours and exposure 4 hours groups, respectively. The tear TNF-αlevel was 901.5±104.5 pg/ml, 1496.2±284.5 pg/ml and 2120.3+718.7 pg/ml in the control, exposure 2 hours and exposure 4 hours groups, respectively. A significant time-dependent change of these three cytokines in tear was found.

**Table 1:** Tear cytokines in different exposure groups. IL = interleukin; TNF = tumor necrosis factor. *p*values were Wilcoxon signed-rank test.

	Post-exposure 0 hour	Post-exposure 2 hours	Post-exposure 4 hours	Post-exposure 0 hour vs. 2 hours (*p* Value)	Post-exposure 0 hourr vs. 4 hours (*p* Value)	Post-exposure 2 hour vs. 4 hours (*p* Value)
IL-8 pg/mL	71.3±10.3	381.5±109.1	631.1±147.5	0.031*	0.031*	0.031*
IL-17 pg/mL	356.2±43.4	342.6±51.6	643.5±159.7	0.22	0.031*	0.031*
TNF-α pg/mL	901.5±104.5	1496.2±284.5	2120.3+718.7	0.063	0.031*	0.063

*p <0.05

## Discussion

Dry eye disease is an important clinical condition that involveschanges of tear film composition, ocular surface damage,corneal dehydration and inflammation.[43–48] In this animal model of short-term desiccation stress (exposure keratopathy), we found that significant corneal epithelial defects(>0.3×0.3 mm^2^per defect area) occurred 2 hours afterexposure, but the central totalcorneal thickness decreased significantly as early as 5 minutes after the insult.Normal corneal stromais composed of layers of well-arranged collagen fibers, and needs to be maintained at approximately78% water content. The intact corneal epithelium has cell-cell tight junctions, and provides the main barrier to prevent corneal stromal dehydration or overhydration.[2, 3]Our findings by in vivo confocal microscopic and SEM revealed the time dependent exfoliation of corneal epithelial cells. The corneal dehydration may occurearlier than epithelial exfoliation and grossly-observable epithelial defect can be detected. The TUNEL staining results demonstrated that corneal epithelial cells underwent apoptosis at 3 hours after air exposure. Although further experiments are needed to confirm the observation, the disruption of the cellular pathophysiology after the induction of short term exposure keratoapthy, which lead to a thinning of corneal stroma, seemed to occurred much earlier.The rapid dehydration of corneal stroma under the induction of exposure keratoapthy underscores the necessity of protecting the corneal surface under any circumstance, and is extremely important for patients undergoingrefractive surgery.Inadequateprotection of the corneal surface from desiccation during surgery for only a limited time period may cause changes incorneal stromal thickness, and thus result inovercorrection after the surgery.[24, 25, 49] Interestingly, our in vivo confocal microscopic results revealed no significant changes in corneal epithelial thickness during the observation process, even though the surface cellswere exfoliated. Therefore, the reduction of the central total cornea thickness was predominantlycaused by dehydration of the corneal stroma instead of corneal epithelial layer.

We also observed the limbal and conjunctival surface, and found that the limbal and conjunctival cells seem to be more resistant to desiccation than corneal cells. The density and morphology of limbal epithelial cells, conjunctival epithelial cells and goblet cells appearsto be relativelytable during the observation periods. We previously demonstrated achange inconjunctival epithelial cells for patients with exposure keratopathy due to Graves' ophthamopathy.[50]Patients with Graves' ophthamopathy suffered from more severe bulbar conjunctival damage and inflammation with the superior site than the temporal site. In a mouse model of desiccation stress,Yoon et al.[51, 52] showed that conjunctival goblet density significantly decreased after 5 days without observing the short-term changes.[52]Our findings demonstrate that goblet cells may exhibitresistance to short-term exposure keratoapthy, which has seldom been emphasized before.

In patients with aqueous deficiency dry eye, the role of inflammation has long been recognized. Immunopathologic changes in the conjunctival epithelium of aqueous tear deficiency dry eye patients includes inflammatory cell infiltration, increased expression of immune activation and adhesion molecules, apoptotic markers, matrix metalloproteinases, inflammatory cytokines, T-helper type 1 (Th-1)attracting chemokines and their receptors.[53–58]However, there are limited results showing the role of inflammation in ocular surface after the induction of exposure keratoapthy.We found thatinflammatory cell infiltration, including AM3K, pan-T, CD4 and CD8 positive cells, occurred as early as 1 hours after exposure. The cells appeared first in the limbal basal epithelial layer, and then migrated time and space-sequentially into the superficial layer of limbal epithelium, the juxtal-limbal corneal and juxta-limbal conjunctival epithelial layer, and the peripheral conjunctiva around 2 mm away from the limbus. At the recovery phase after termination of eye exposure for 4 hours, the significant infiltration of the inflammatory cells can still be detected on limbus and juxta-limbal conjunctiva for at least 8 hours. Such finding implied the slow recovery of the inflammatory response after termination of eye exposure. Our in vivo confocal microscopic and SEM findings also demonstrated a time depended recovery of corneal surface epithelial cells after termination of eye exposure (data not shown).In general, the in vivo confocal microscopic findings revealed more information than impression cytologic findings, since the latter only collected the superficial layer of the ocular surfaceafter mechanical peeling. The results from impression cytology may only provide limited information.Our results pointed out the origin of the inflammatory cells is from limbal vessels instead of the tear film, and the infiltration of inflammatory cells reached perilimbal corneal epithelium no less than 4 hours after exposure. Therefore, in the clinical practice, we must be vigilantwith regard to cornealprotection in patients with exposure keratopathy, especially forpotential corneal donors.The infiltration of inflammatory cells in exposure keratopathy is important forclinical differentiation between infectious and non-infectious keratitis. The proper diagnosis willinfluence the treatment strategy, and the decision of qualifying potential donor corneasfor further transplantation. Since the sterile infiltration of the inflammatory cells may mimic infectious keratitis, the decision of initiating antibiotic treatment in patients, and the qualification of donor corneas without microbiological proofwould be difficult.

Our immunohistochemical results demonstrated the inflammatory cells that infiltrated after the induction of exposure keratopathy were AM3K(+), Pan-T(+), CD4(+) and CD8(+) cells. T helper cellshavelong been known to play a key role in aqueous tear deficiency. However, to the best of our knowledge, the role of macrophages/neutrophils and T killer cells in dry eye has not been reported previously. It has been recognized that the inflammatory mediators associated with aqueous tear deficiency dry eye included Th1-related cytokines (e.g INF-alpha), Th17-related cytokines, chemokines and theirreceptors, metalloproteinase, and secretory phospholipases (e.g IL-1β, IL-6, IFN-γ and TNF-α) and MMPs.[57, 59–63]We analyzed the tear cytokines (IL-8, IL-17 and TNF-α), and found a time dependent increase of these three cytokines after exposure, which was correlated to the time dependent change of the inflammatory cellular infiltration revealed by in vivo confocal microscopy. IL-8 is a potential neutrophil chemotactic/activation factor. It is a primer inflammatory cytokine secreted responding to proinflammatory stimuli in many cell types, including macrophages, T-cells, neutrophils, monocytes, fibroblasts, and endothelial cells. [64] IL-8 is also secreted from corneal epithelial cells and retinal cells. [65] IL-17 is produced by T helper type lymphocyte, and has been studied as a possible connection between inflammation and disruption of the corneal barrier after desiccating stress. [58, 66–67] TNF-α is a pleiotropic cytokine that has multiple proinflammatory and costimulatory effects on a broad range of cell types. [68]Ji YW, et al demonstrated that neutralization of ocular surface TNF-α reduces ocular surface and lacrimal gland inflammation induced by in vivo dry eye.[69] Our result demonstrated a mixture of cytokines involved in the exposure keratopathy. It is important to further identify the detailed expression of the inflammatory cytokines, and the difference between aqueous tear deficiency and exposure keratoapthy.

There are some limitations of this study.First of all, initially we attemptedto mimic evaporative dry eye, a chronic pathophysiology process, the study design more closely approximates acute pathophysiology process of exposure keratopathy found in intensive care unit patients, or those under general anaesthesia. In those patients, poor eyelid closure and decreased blink reflex for more than several hours or even several days may cause conjunctival hyperemia, mucopurelent secretion, corneal staining, corneal filaments and infectious keratitis,[16, 17, 70] which are different from the signs and symptoms of other patients with evaporative dry eye experience.[71] Second, *in vivo*confocal microscopy only revealed morphological changes in the observed cells and structures. Immunohistochemistry and other methods may be needed for further identification the changes of cellular micro-structure of the ocular surface cells. Third, the rabbit model is different from the human corneas for have no Bowman's layer. The hematologic profile is also not similar between these two species. However, our study is still valuablefrom several aspects. Weperformed a multimodal evaluation of the ocular surface corneal and conjunctival changes in exposure keratopathy, and provide information that has not been previously reported.

In conclusion, this study provides a successful model of exposure keratopathy.We found changesin central totalcorneal thickness, morphological changesto epithelial cells,inflammatory cell infiltration on ocular surface and change of several tear cytokines no less than 4 hours after exposure. These finding pointed out the vulnerability of ocular surface under a short-term exposure keratopathy.
